# Integrative transcriptomic and proteomic profiling of the effects of cell confluency on gene expression

**DOI:** 10.1038/s41597-024-03465-z

**Published:** 2024-06-12

**Authors:** Vivian Lobo, Evgeniia Shcherbinina, Jakub O. Westholm, Iwona Nowak, Hsiang-Chi Huang, Davide Angeletti, Dimitrios G. Anastasakis, Aishe A. Sarshad

**Affiliations:** 1 Department of Medical Biochemistry and Cell biology, Institute of Biomedicine, University of Gothenburg, SE 40530 Gothenburg, Sweden; 2 Wallenberg Centre for Molecular and Translational Medicine, University of Gothenburg, SE 40530 Gothenburg, Sweden; 3 Department of Biochemistry and Biophysics, National Bioinformatics Infrastructure Sweden, Science for Life Laboratory, Stockholm University, Box 1031, SE 17121 Solna, Sweden; 4 Department of Microbiology and Immunology, Institute of Biomedicine, University of Gothenburg, SE 40530 Gothenburg, Sweden; 5 SciLifeLab, Institute of Biomedicine, University of Gothenburg, SE 40530 Gothenburg, Sweden; 6 RNA Molecular Biology Laboratory, National Institute for Arthritis and Musculoskeletal and Skin Disease, Bethesda, MD 20892 USA

**Keywords:** Cell biology, Cancer

## Abstract

In this study we examine the impact of cell confluency on gene expression. We focused on Argonaute (AGO) protein dynamics and associated gene and protein expression in HEK293, A375, and SHSY5Y cell lines. As a consequence of cell confluency, AGO2 protein translocates into the nucleus. Therefore, we generated transcriptomic data using RNA sequencing to compare gene expression in subconfluent versus confluent cells, which highlighted significant alterations in gene regulation patterns directly corresponding to changes in cell density. Our study also encompasses miRNA profiling data obtained through small RNA sequencing, revealing miRNA expressional changes dependent on cellular confluency, as well as cellular localization. Finally, we derived proteomic data from mass spectrometry analyses following AGO1-4 immunoprecipitation, providing a comprehensive view of AGO interactome in both nuclear and cytoplasmic compartments under varying confluency. These datasets offer a detailed exploration of the cellular and molecular dynamics, influenced by cell confluency, presenting a valuable resource for further research in cellular biology, particularly in understanding the basic mechanisms of cell density in cancer cells.

## Background & Summary

Cell confluency, defined as the measurement of cell density in a culture dish or flask that corresponds to the percentage of the surface area covered by adherent cells^1^, is one of the crucial factors that affects the characteristics of tumor cells in cell culture^2^. Increase in cell density intensifies intracellular interactions and influences cellular metabolism^3^ and expression of genes and proteins^2^. Notably, it has been shown that cell confluence correlates with glucose uptake and lactate production in epithelial cells^3^ and affects gene expression^4,5^. Studies of changes in cellular morphology, physiology and molecular traits at different levels of cell confluency can make a great impact to uncovering basic cellular mechanisms and allows for enhanced conditions in cell culture work based on the needs of the experiment^6^. In particularly, contact inhibition – the process which leads to cell growth arrest when cells form a monolayer in culture dish and come in contact with each other^7^ – is actively studied in cancer cells^8,9^. Reaching 100% confluence, normal healthy cells stop proliferating, while cancer cells do not have a contact inhibition mechanism and continue to divide^7^.

Several studies showed that microRNA (miRNA) biogenesis can be regulated in a cell-density-dependent manner via different pathways^10,11^. For example, Mori *et al*. described that at high cell density the tumor-suppressive Hippo pathway is active and prevents binding of YAP to its downstream target p27, which is a regulatory component of the miRNA-processing machinery. This results in a widespread miRNA repression and leads to the activation of MYC expression observed in cancer cells^10^. Moreover, Hwang *et al*. demonstrated a global activation of miRNA biogenesis in mammalian and *Drosophila* cells grown at high confluency^11^. miRNAs are short (18–24 nt) non-coding RNA molecules that post-transcriptionally regulate the expression of target messenger RNAs (mRNAs) via RNA interference (RNAi) pathways^11^. The guide strand of miRNAs binds to the RNA-induced silencing complex (RISC), which then recognizes the complementary sequences of 3′ untranslated region (3′UTR) of target mRNAs. The main component of RISC are the Argonaute (AGO) proteins, which facilitates the recognition of target mRNA and subsequent target silencing^12^.

Human cells express four AGO proteins (AGO1-4) in a tissue-specific manner. AGO1 and AGO2 are better studied than the other members of the AGO protein family and have more prominent expression^13^. During the early stages of embryonic development the ratio of different AGO proteins are tightly controlled^13^ and the loss of AGO2 function leads to embryonic lethality^14^. For a long time AGO2 was thought to be the only member of the AGO protein family which had the capability to cleave target mRNA^15^. However, recently Park *et al*. showed that when bound to particular miRNAs AGO3 can also slice target RNAs^16^.

RNAi, mediated by the RISC complex, has mainly been considered to be executed in the cytoplasm, but a large body of work has convincingly shown that AGO proteins can be localized in the nucleus of some cells^17^ or translocate there under certain conditions^18^. Recently, Johnson *et al*. showed that increased cell confluency promotes AGO2 nuclear localization in HCT116 colon cancer cells^12^. They observed that cells grown up to 300–400% confluency had a significant increase in AGO2 nuclear levels compared to cells grown at 50–100% confluency. Evaluation of the miRNA expression in HCT116 grown at high cell density revealed a global increase in miRNAs biogenesis. Moreover, nuclear localization of AGO2 attenuated the repression of target mRNAs by miRNAs in cytoplasm^12^.

**Fig. 1:**
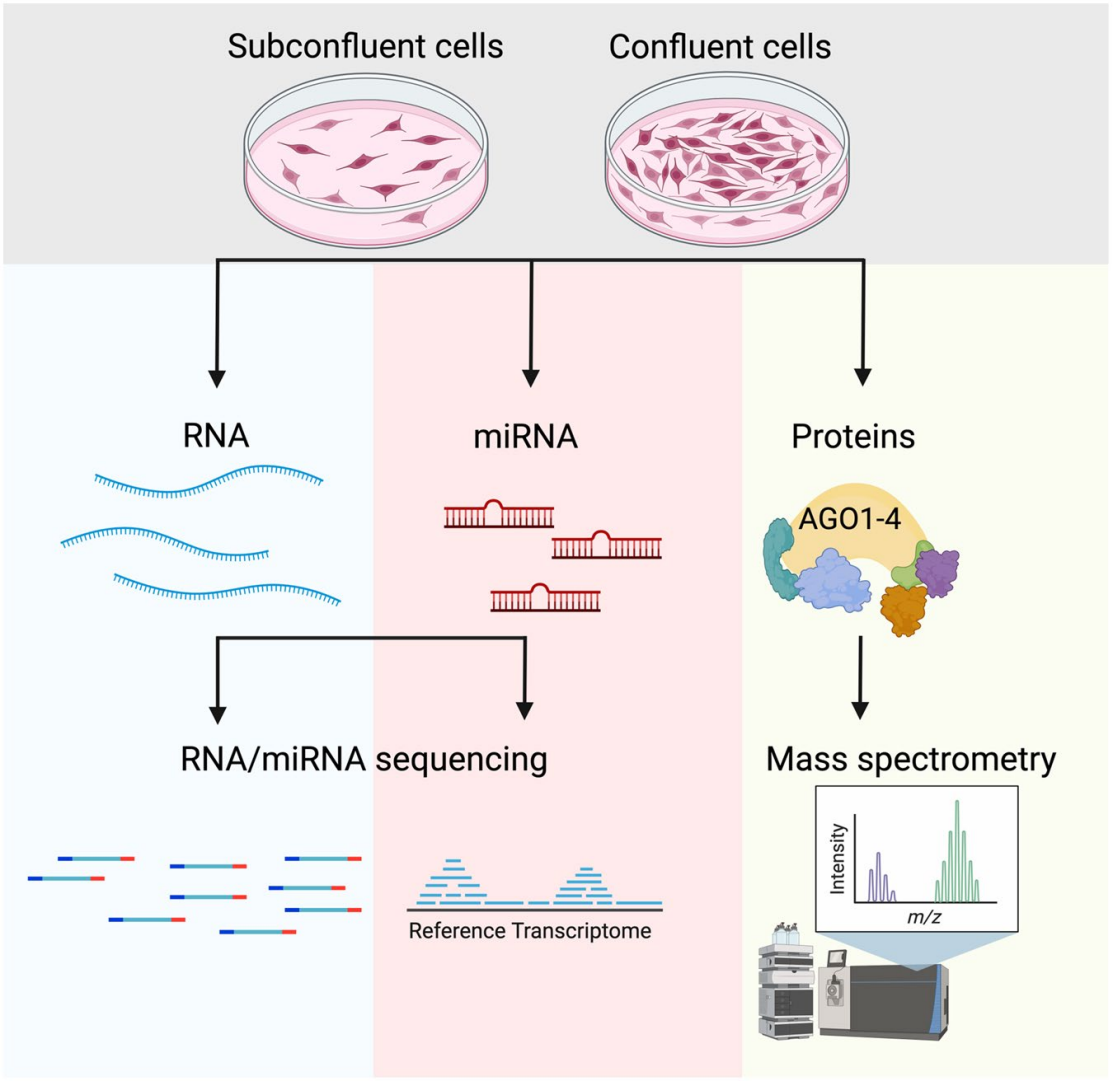
Schematic representation of the workflow outlined in this study.

In this study we aimed to describe the transcriptomic and proteomic profiling of AGO1-4 dynamics at different cell confluency in the embryonic kidney HEK293 cell line, the melanoma cancer cell lines A375 and in the neuroblastoma cell line SHSY5Y (Fig. 1). To define differences in the gene expression landscape between subconfluent and confluent cells, we performed RNA sequencing experiments in all three cell lines. Since AGO proteins are key mediators of miRNA action, we performed small RNA sequencing and profiled the miRNA populations in subconfluent and confluent conditions. Finally, to access the interactome of AGO1-4 in nucleus and cytoplasm in confluent and subconfluent cells we performed mass spectrometry analysis of cell lysates after AGO immunoprecipitation. Our collective data indicates that cell confluency affects global gene expression, which may in turn be linked to AGO2 translocation into the nucleus in confluent cells.

## Methods

### Cell culture

**Fig. 2:**
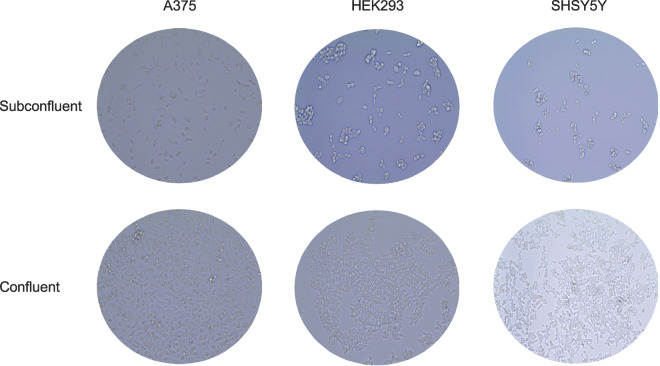
Subconfluent and confluent HEK293, A375 and SHSY5Y cells. Microscopy images of subconfluent and confluent HEK293, A375 and SHSY5Y cells used in this study.

Experiments were performed using human embryonic kidney HEK293 (ATCC, CRL-1573), human melanoma A375 (ATCC, CRL-1619) and human neuroblastoma SHSY5Y (ATCC, CRL-2266) cell lines. HEK293 and A375 cells were cultured in Dulbecco’s modified Eagle’s medium (DMEM) (Gibco, 11995065), supplemented with 10% fetal bovine serum (FBS) (Gibco, 11573397) and 100 U/ml penicillin-streptomycin (Gibco, 11548876) in a humidified incubator at 37°C and 5% CO2. SHSY5Y cells were cultured in Roswell Park Memorial Institute (RPMI) 1640 media (61870010, Gibco) supplemented with 10% FBS (Gibco, 11573397) and 100 U/ml penicillin-streptomycin (Gibco, 11548876) in a humidified incubator at 37°C and 5% CO2. Cells were considered as subconfluent and confluent (also herein referred to as overconfluent) when they reached 40–50% and 75–100% of confluency, respectively (Fig. 2). We previously showed that cancer cell lines differ in the level of nuclear AGO2 protein: some almost completely lack nuclear AGO2, while others showed an even distribution of AGO2 between cytoplasmic and nuclear fractions^17^. For this study we chose HEK293, A375 and SHSY5Y cells, because under subconfluent conditions these cell lines have AGO2 located predominantly in the cytoplasm^17^.

### Biochemical fractionation

Biochemical fractionation of cells was done as previously described by Gagnon *et al*.^19^ with following changes^20^.Briefly, cell pellets were gently resuspended in a hypotonic lysis buffer (10 mM Tris-HCl, pH 7.6, 10 mM NaCl, 3 mM MgCl_2_, 0.3% NP-40 and 10% Glycerol), supplemented with a protease inhibitor cocktail, and then centrifugated for 2 minutes at 200 g. The supernatant was collected, cleared with centrifugation at 12000 *g* for 20 minutes and stored as the cytoplasmic fraction. The remaining nuclear pellet was washed 3 times with the hypotonic lysis buffer and collected by centrifugation for 2 minutes at 200 *g*. Each time the supernatant was discarded. The nuclear fraction was obtained from the remaining pellet, using a nuclear lysis buffer (20 mM Tris-HCl, pH 7.6, 150 mM KCl, 3 mM MgCl_2_, 0.3% NP-40, 10% Glycerol) supplemented with protease inhibitor cocktail. The lysate was sonicated twice for 10 seconds at 60% amplitude (Sonics, VCX130). The nuclear fraction was cleared with centrifugation at 12000 *g* for 20 minutes and the supernatant collected. Protein concentration for cytoplasmic fraction was measured using Bradford Reagent (B6916, Sigma Aldrich). Calreticulin and Lamin A/C are used to show the purity of cytoplasmic and nuclear fractions, respectively.

### Western blotting

For Western blotting analysis 5–20 μg of cytoplasmic and nuclear protein samples were run on 4–12% Bis-Tris gels and transferred to nitrocellulose membrane (Cytiva, 1060000) using semi-dry transfer unit TE70X (Hoefer Inc.). Membrane was blocked with 2% milk for 1 h at room temperature and the proteins of interest were analyzed by hybridization with primary antibodies: anti-AGO2 (Abcam, ab32381, 1:4000), anti-Calreticulin (Santa Cruz, sc-373863, 1:1000), anti-Lamin A/C (Santa Cruz, sc-376248, 1:4000). For protein visualization we used appropriate HRP-linked secondary antibodies (for AGO2) and Thermo Scientific SuperSignal™ West Dura Extended Duration Substrate (ThermoFisher, 34076). Membranes were visualized using ChemiDoc MP imaging system (Bio-Rad Laboratories, 12003154).

### RNA sequencing

Total RNA was extracted from HEK293, A375 and SHSY5Y using the Quick-RNA Miniprep Kit (ZYMO Research) following the manufacturer’s protocol. The RNA concentration and quality were analyzed using Agilent 2200 TapeStation System. RNA samples with RNA Integrity Number higher than 8 were sent to SNP&SEQ Technology Platform (NGI Uppsala, Sweden). Libraries were prepared from 300 ng RNA using the Illumina Stranded Total RNA library preparation kit, including Ribo-Zero Plus treatment (20040525/20040529, Illumina Inc.) according to manufacturer’s instructions. For indexing Unique Dual Indexes (20040553/20040554, Illumina Inc.) were used. Sequencing was carried out with NovaSeq 6000 system using paired-end 150 bp read length, S4 flowcell and v1.5 sequencing chemistry. RNA-seq data were pre-processed using the RNA-seq nf-core pipeline^21^. Differential expression analysis was done using DEseq. 2^22^, on genes with at least 10 reads in at least 3 samples. Genes with FDR adjusted p-value < 0.01 and absolute log2 fold change >0.5 were considered differentially expressed.

### Immunoprecipitation assays and AGO protein affinity purification with T6B peptide

For identification of AGO1,2,3,4-interacting protein partners and miRNAs, we used Flag-tagged T6B peptide for AGO1-4 isolation^23^. Volumes corresponding to 3 mg of cytoplasmic fraction were used together with 400 μg of T6B peptide. Anti-Flag M2 beads (M8823, Millipore) were conjugated with T6B peptide for 4 h, washed and incubated with protein lysates. Next, beads were washed with NP-40 buffer. T6B bound proteins were eluted by incubation with 0.2 M Glycine, pH 2.5 by gentle shaking for 15 min followed by neutralization of the eluate using 1 M Tris, pH 8. The pull-down efficiency was confirmed by western blot and the eluate submitted for mass spectrometry analysis at Proteomics Core Facility (University of Gothenburg, Sweden). For miRNAs, TRIzol was added directly to the beads after AGO1-4 immunoprecipitation and the RNA was isolated using manufacturer’s instructions. The extracted RNA was taken through the small RNA library preparation outlined below.

### miRNA sequencing

miRNAs were isolated using TRIzol reagent according to the manufactures instructions and converted into libraries for sequencing as previously described^24^ with a few modifications. Briefly, purified miRNAs were subject’ed to 3′ adapter ligation with 5′-adenylated DNA adapter (see 3′ adapters in table below) using Rnl2(1–249)K227Q RNA ligase (NEB) according to the manufacturer’s instructions at 4 °C overnight. Ligated RNA was pooled and purified using oligo clean and concentrate kit (ZYMO Research) and subjected to 5′ adapter ligation with a 5′ chimeric DNA-RNA adapter (5’aminolinker-GTTCAGAGTTCTACAGTCCGACGATCrNrNrNrN) using RNA ligase (Thermo Fisher Scientific) at 37 °C for 1 hour. The 3′- 5′- ligated RNA was purified using oligo clean and concentrate kit (ZYMO Research) and subjected to reverse transcription using SuperScript® IV (200 U/µl, Thermo Fisher Scientific) according to the manufacturer’s instructions using RT primer (GCCTTGGCACCCGAGAATTCCA). The cDNA was amplified using Platinum Taq DNA Polymerase (Thermo Fisher Scientific), according to the manufacturer’s instructions using 5′-medium PCR primer (CTCTACACGTTCAGAGTTCTACAGTCC) and 3′ medium PCR primer (CCTGGAGTTCCTTGGCACCCGAGAATT) for 6 cycles. Then the PCR product was purified using the DNA Clean & Concentrator™-5 kit (ZYMO Research), eluted with 32 µl of nuclease free water, and size selected (74–88 bp) using 3% agarose Pippin Prep (Sage Science). Following size selection, a second round of (X cycle) PCR was performed using the same polymerase, a 5′-long PCR primer: (AATGATACGGCGACCACCGAGATCTACACGTTCAGAGTTCTACAGTCCGA), and 3′ indexed primer (see 3′ index primers in Table 1), Libraries were sequenced on a Illumina NovaSeq 6000. Bcl files were converted to fastq files using bcl2fastq. Adapters were trimmed using cutadapt v 2.4. and reads were mapped to the human miRNAs using bowtie2^25^.

**Table 1:** List of 3′ index primers for miRNA sequencing.

Sample	Illumina Index primer	3′ adapter
HEK293 sub1	CAAGCAGAAGACGGCATACGAGATGGTGTCTTGTGACTGGAGTTCCTTGGCACCCGAGAATTCCA	5′-rAppNNCAGCATTGGAATTCTCGGGTGCCAAGG-L
HEK293 sub2		5′-rAppNNATAGTATGGAATTCTCGGGTGCCAAGG-L
HEK293 sub3		5′-rAppNNTCATAGTGGAATTCTCGGGTGCCAAGG-L
HEK293 OC1	CAAGCAGAAGACGGCATACGAGATTCAACTGGGTGACTGGAGTTCCTTGGCACCCGAGAATTCCA	5′-rAppNNCAGCATTGGAATTCTCGGGTGCCAAGG-L
HEK293 OC2		5′-rAppNNATAGTATGGAATTCTCGGGTGCCAAGG-L
HEK293 OC3		5′-rAppNNTCATAGTGGAATTCTCGGGTGCCAAGG-L
**miRNApool1**		
SHSY5YSubC1	CAAGCAGAAGACGGCATACGAGATCTCCTAGAGTGACTGGAGTTCCTTGGCACCCGAGAATTCCA	5′-rAppNNTAGCGATGGAATTCTCGGGTGCCAAGG-L
SHSY5YsubN1		5′-rAppNNCTGTAGTGGAATTCTCGGGTGCCAAGG-L
SHSY5YsubC2		5′-rAppNNTAGTCGTGGAATTCTCGGGTGCCAAGG-L
SHSY5YsubN2		5′-rAppNNAGTGTCTGGAATTCTCGGGTGCCAAGG-L
SHSY5YsubC3		5′-rAppNNATCGACTGGAATTCTCGGGTGCCAAGG-L
SHSY5YsubN3		5′-rAppNNGACATGTGGAATTCTCGGGTGCCAAGG-L
SHSY5YoverC1		5′-rAppNNGACTACTGGAATTCTCGGGTGCCAAGG-L
SHSY5YoverN1		5′-rAppNNATCTCGTGGAATTCTCGGGTGCCAAGG-L
SHSY5YoverC2		5′-rAppNNTCTGTGTGGAATTCTCGGGTGCCAAGG-L
SHSY5YoverN2		5′-rAppNNCAGCATTGGAATTCTCGGGTGCCAAGG-L
SHSY5YoverC3		5′-rAppNNATAGTATGGAATTCTCGGGTGCCAAGG-L
SHSY5YoverN3		5′-rAppNNTCATAGTGGAATTCTCGGGTGCCAAGG-L
**miRNApool2**		
A375SubC1	CAAGCAGAAGACGGCATACGAGATTAGTTGCGGTGACTGGAGTTCCTTGGCACCCGAGAATTCCA	5′-rAppNNTAGCGATGGAATTCTCGGGTGCCAAGG-L
A375SubN1		5′-rAppNNCTGTAGTGGAATTCTCGGGTGCCAAGG-L
A375SubC2		5′-rAppNNTAGTCGTGGAATTCTCGGGTGCCAAGG-L
A375SubN2		5′-rAppNNAGTGTCTGGAATTCTCGGGTGCCAAGG-L
A375SubC3		5′-rAppNNATCGACTGGAATTCTCGGGTGCCAAGG-L
A375SubN3		5′-rAppNNGACATGTGGAATTCTCGGGTGCCAAGG-L
A375OverC1		5′-rAppNNGACTACTGGAATTCTCGGGTGCCAAGG-L
A375OverN1		5′-rAppNNATCTCGTGGAATTCTCGGGTGCCAAGG-L
A375OverC2		5′-rAppNNTCTGTGTGGAATTCTCGGGTGCCAAGG-L
A375OverN2		5′-rAppNNCAGCATTGGAATTCTCGGGTGCCAAGG-L
A375OverC3		5′-rAppNNATAGTATGGAATTCTCGGGTGCCAAGG-L
A375OverN3		5′-rAppNNTCATAGTGGAATTCTCGGGTGCCAAGG-L
**miRNApool3**		
HEK293SubC1	CAAGCAGAAGACGGCATACGAGATGAGATACGGTGACTGGAGTTCCTTGGCACCCGAGAATTCCA	5′-rAppNNTAGCGATGGAATTCTCGGGTGCCAAGG-L
HEK293SubN1		5′-rAppNNCTGTAGTGGAATTCTCGGGTGCCAAGG-L
HEK293SubC2		5′-rAppNNTAGTCGTGGAATTCTCGGGTGCCAAGG-L
HEK293SubN2		5′-rAppNNAGTGTCTGGAATTCTCGGGTGCCAAGG-L
HEK293SubC3		5′-rAppNNATCGACTGGAATTCTCGGGTGCCAAGG-L
HEK293SubN3		5′-rAppNNGACATGTGGAATTCTCGGGTGCCAAGG-L
HEK293OverC1		5′-rAppNNGACTACTGGAATTCTCGGGTGCCAAGG-L
HEK293OverN1		5′-rAppNNATCTCGTGGAATTCTCGGGTGCCAAGG-L
HEK293OverC2		5′-rAppNNTCTGTGTGGAATTCTCGGGTGCCAAGG-L
HEK293OverN2		5′-rAppNNCAGCATTGGAATTCTCGGGTGCCAAGG-L
HEK293OverC3		5′-rAppNNATAGTATGGAATTCTCGGGTGCCAAGG-L
HEK293OverN3		5′-rAppNNTCATAGTGGAATTCTCGGGTGCCAAGG-L

### Proteomic analysis, sample preparation and digestion

The samples were processed using modified filter-aided sample preparation (FASP) method^26^. In short, samples were reduced in 100 mM dithiothreitol at 56 °C for 30 min, transferred to Microcon-Biomax membrane 30 kDa Centrifugal Filter Units (Merck), washed several times with 8 M urea and once with digestion buffer (DB, 50 mM TEAB, 0.5% sodium deoxycholate (SDC)) prior to alkylation with 375 mM iodoacetamide in DB for 30 min in room temperature. Samples were digested with trypsin (Pierce MS grade Trypsin, Thermo Fisher Scientific, ratio 1:50) at 37 °C overnight and an additional portion of trypsin was added and incubated for another three hours. Peptides were collected by centrifugation and labelled using TMTpro 18-plex isobaric mass tagging reagents (Thermo Fisher Scientific) according to the manufacturer instructions. The samples were combined into one TMT-set and SDC was removed by acidification with 10% TFA. The TMT-set was purified using HiPPR detergent removal kit (Thermo Scientific) and Pierce peptide desalting spin columns (Thermo Scientific), according to the manufacturer´s instructions prior to basic reversed-phase chromatography (bRP-LC) fractionation. Peptide separation was performed using a Dionex Ultimate 3000 UPLC system (Thermo Fischer Scientific) and a reversed-phase Xbridge BEH C18 column (3.5 μm, 3.0 × 150 mm, Waters Corporation) with a gradient from 3% to 100% acetonitrile in 10 mM ammonium formate at pH 10.00 over 22 min at a flow of 400 µL/min. The 20 fractions were concatenated into 10 fractions, dried and reconstituted in 3% acetonitrile, 0.1% trifluoric acid.

### NanoLC-MS/MS analysis and database search

Each fraction was analysed on Orbitrap Lumos™ Tribrid™ mass spectrometer equipped with the FAIMS Pro ion mobility system interfaced with nLC 1200 liquid chromatography system (all Thermo Fisher Scientific). Peptides were trapped on an Acclaim Pepmap 100 C18 trap column (100 μm x 2 cm, particle size 5 μm, Thermo Fischer Scientific) and separated on an in-house constructed analytical column (370 × 0.075 mm I.D.) packed with 3 μm Reprosil-Pur C18-AQ particles (Dr. Maisch, Germany) using a gradient from 3% to 80% acetonitrile in 0.2% formic acid over 90 min at a flow of 300 nL/min. FAIMS Pro was alternating between the compensation voltages (CV) of −50 and −70, and essentially the same data-dependent settings were used at both CVs. Precursor ion mass spectra were acquired at 120 000 resolution, scan range 375–1375 and maximum injection time 50 ms. MS2 analysis was performed in a data-dependent mode, where the most intense doubly or multiply charged precursors were isolated in the quadrupole with a 0.7 m/z isolation window and dynamic exclusion within 10 ppm for 60 s. The isolated precursors were fragmented by collision induced dissociation (CID) at 35% collision energy with the maximum injection time of 35 ms for 3 s (‘top speed’ setting) and detected in the ion trap, followed by multinotch (simultaneous) isolation of the top 10 MS2 fragment ions within the m/z range 400–1400, fragmentation (MS3) by higher-energy collision dissociation (HCD) at 55% collision energy and detection in the Orbitrap at 50 000 resolution m/z range 100–500 and maximum injection time 105 ms.

The data files for the set were merged for identification and relative quantification using Proteome Discoverer version 2.4 (Thermo Fisher Scientific). The search was against Swissprot human database (May 2022) using Sequest as a search engine with precursor mass tolerance of 10 ppm and fragment mass tolerance of 0.6 Da. Tryptic peptides were accepted with zero missed cleavage, variable modifications of methionine oxidation and fixed cysteine alkylation, TMTpro-label modifications of N-terminal and lysine were selected. Percolator was used for PSM validation with the strict FDR threshold of 1%. TMT reporter ions were identified with 3 mmu mass tolerance in the MS3 HCD spectra. Only the quantitative results for the unique peptide sequences with the minimum SPS match % of 65 and the average S/N above 10 were taken into account for the protein quantification. The quantified proteins were filtered at 5% FDR and grouped by sharing the same sequences to minimize redundancy.

## Data Records

The mass spectrometry proteomics data have been deposited to the ProteomeXchange Consortium via the PRIDE partner repository^27^ with the dataset identifier PXD047707^28^. RNA-seq data are available on the NCBI Short-Read Archive (SRA) under the accession number GSE249290^29^. The miRNA-seq data are available on the NCBI Short-Read Archive (SRA) under the accession number GSE255251^30^.

## Technical Validation

### Subcellular localization of AGO2 protein in cancer cells under different confluency conditions

**Fig. 3:**
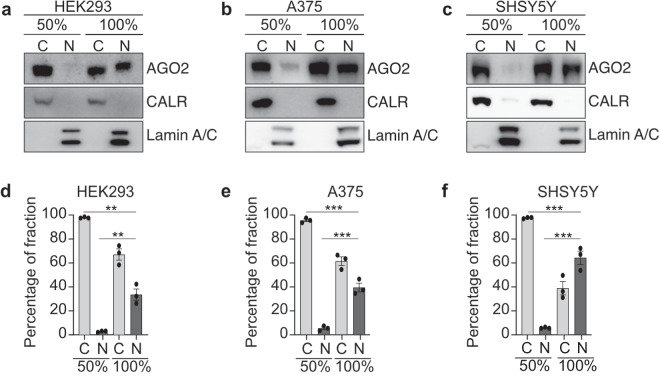
AGO2 subcellular localization in subconfluent and confluent HEK293, A375 and SHSY5Y cells. Representative AGO2 immunoblots from cytoplasmic and nuclear fractions of subconfluent (50%) and confluent (100%) (**a**) HEK293 cells, (**b**) A375 cells and (**c**) SHSY5Y cells, showing AGO2 distribution between nuclear and cytoplasmic fractions. Quantification of AGO2 distribution between the cytoplasmic and nuclear fractions in subconfluent (50%) and confluent (100%) (**d**) HEK293 cells, (**e**) A375 cells and (**f**) SHSY5Y cells. Calreticulin (CALR) and Lamin A/C serve as a cytoplasmic and nuclear markers, respectively.

We previously demonstrated that AGO2 is dynamically distributed between the cytoplasmic and nuclear fraction in various cancer cells^17^. Some cancers are characterized by ubiquitous distribution of AGO2 while other cancer cells completely lack AGO2 from the nucleus^17^. To investigate AGO2 subcellular localization in subconfluent and confluent HEK293, A375 and SHSY5Y cells we performed a western blot immunoassay for cytoplasmic and nuclear fractions (Fig. 3a-c) and showed that cancer cells can increase their pool of nuclear AGO2 distribution directly as a measure of cell confluence (Fig. 3a-c). Subconfluently grown HEK293, SHSY5Y and A375, which completely lack AGO2 in the nucleus, become nuclear AGO2 positive when cells are grown to confluency (Fig. 3a-c). In HEK293 cells we observed a 31% increase in the amount of nuclear AGO2, while for A375 and SHSY5Y the increase was 34% and 57%, respectively (Fig. 3d-f).

### Quality assessment of RNA sequencing data obtained for subconfluent and confluent cancer cells

**Fig. 4:**
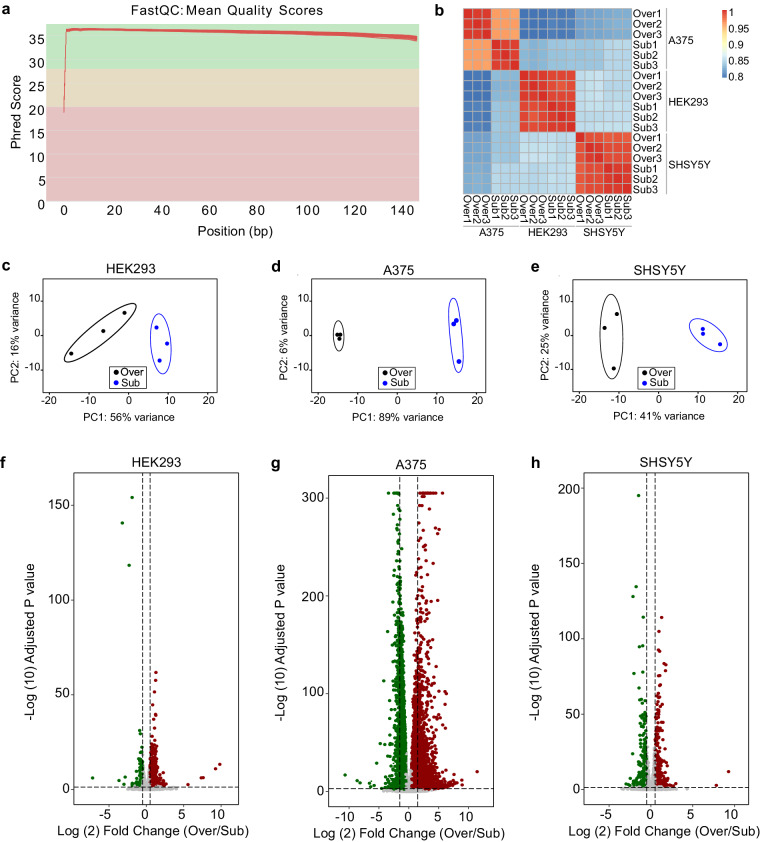
RNA sequencing data for subconfluent and confluent HEK293, A375 and SHSY5Y. (**a**) RNA sequencing quality metrics depicting overall quality per sequence, with FastQC^31^. The x-axis represents the base position in the read, while the y-axis depicts the Phred quality score. (**b**) Heatmap of correlation matrix of RNA-seq for confluent and subconfluent HEK293, A375, and SHSY5Y cancer cell lines. Color intensity indicates correlation coefficients. Principal component analysis (PCA) of RNA sequencing samples in (**c**) HEK293 cells, (**d**) A375 cells and (**e**) SHSY5Y cells shows samples clustering according to cells confluency status. Volcano plots of RNA sequencing data illustrating the differential gene expression between subconfluent and confluent (**f**) HEK293 cells, (**g**) A375 cells and (**h**) SHSY5Y cells. Each gene is plotted based on its fold change and the statistical significance is represented as -log10(p-value). Genes exhibiting significant upregulation in confluent conditions are depicted in red, while those downregulated are shown in green.

To gain insights into the transcriptional landscape and potential effect on gene expression that may be mediated by cellular confluency, we performed RNA sequencing (RNA-seq) experiment in HEK293, SHSY5Y and A375 cells in three replicates. Cells were grown to subconfluency or confluency and RNA was extracted. Pure RNA was used to prepare sequencing libraries using Illumina Stranded Total RNA Prep in conjunction ribosome depletion. The reads were mapped to the human genome (GRCh38) and each sample contained more than 100 million reads (Table S1). On average we observed about 40% of sequencing duplicates, which indicates high sequencing depths. The percentages of successfully mapped unique reads varied between 80,87% and 89,47% (Table S1). Furthermore, FastQC plots revealed sufficient quality scores for all reads, which indicates robust data quality for further analysis (Fig. 4a). Heatmap of correlation matrix of RNA-seq samples shows the correlation coefficients distribution from 0.8 to 1 (Fig. 4b). The principal component analysis (PCA) showed that RNA-seq samples are clustered according to their confluency status in all three cell lines (Fig. 4c-e). Finally, we analyzed the differential gene expression in the respective cell lines. Using a cutoff of 1.5 logarithmic (log2) fold change and a p-value < 0.05, we found 757 differentially upregulated and 178 downregulated genes in HEK293 cells (Fig. 4f; Table S2), 459 upregulated and 362 downregulated genes in SHSY5Y cells (Fig. 4g; Table S3) and finally we identified 4020 differentially upregulated and 3375 downregulated genes in A375 cells (Fig. 4h; Table S4).

### miRNA sequencing from cytoplasmic and nuclear fraction of subconfluent and confluent cancer cells

**Fig. 5:**
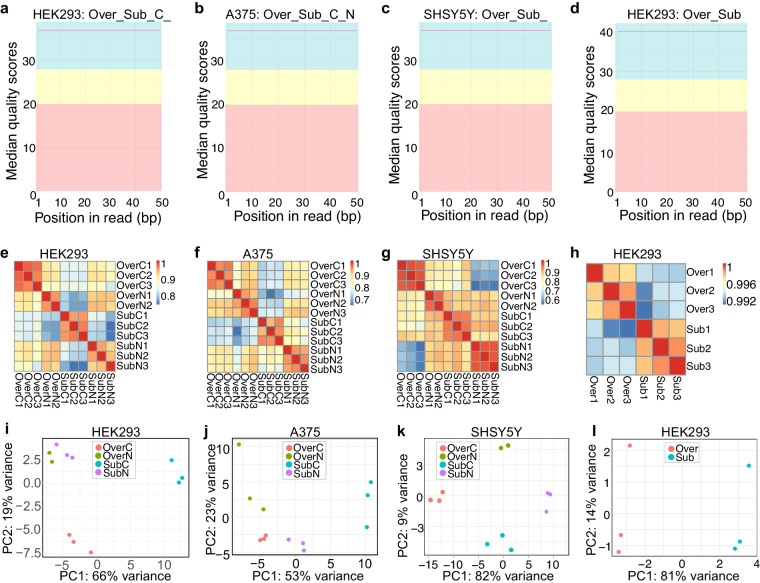
miRNA sequencing data for subconfluent and confluent HEK293, A375 and SHSY5Y. RNA sequencing quality metrics depicting overall quality per sequence, with FastQC^31^, in fractionated (**a**) HEK293, (**b**) A375, (**c**) SHSY5Y cells and (**d**) total HEK293 cells after T6B immunoprecipitation. T6B peptide was used for AGO1-4 proteins pull down. The x-axis represents the base position in the read, while the y-axis depicts the quality score. Heatmap of correlation matrix of miRNA-seq for confluent and subconfluent fractionated (**e**) HEK293, (**f**) A375, (**g**) SHSY5Y cells and (**h**) HEK293 cells after T6B immunoprecipitation. Color intensity indicates correlation coefficients. Principal component analysis (PCA) of miRNA-seq samples in (**i**) HEK293, (**j**) A375, (**k**) SHSY5Y cells and (**l**) HEK293 cells after T6B immunoprecipitation shows samples clustering according to cells confluency status and subcellular localization.

**Fig. 6:**
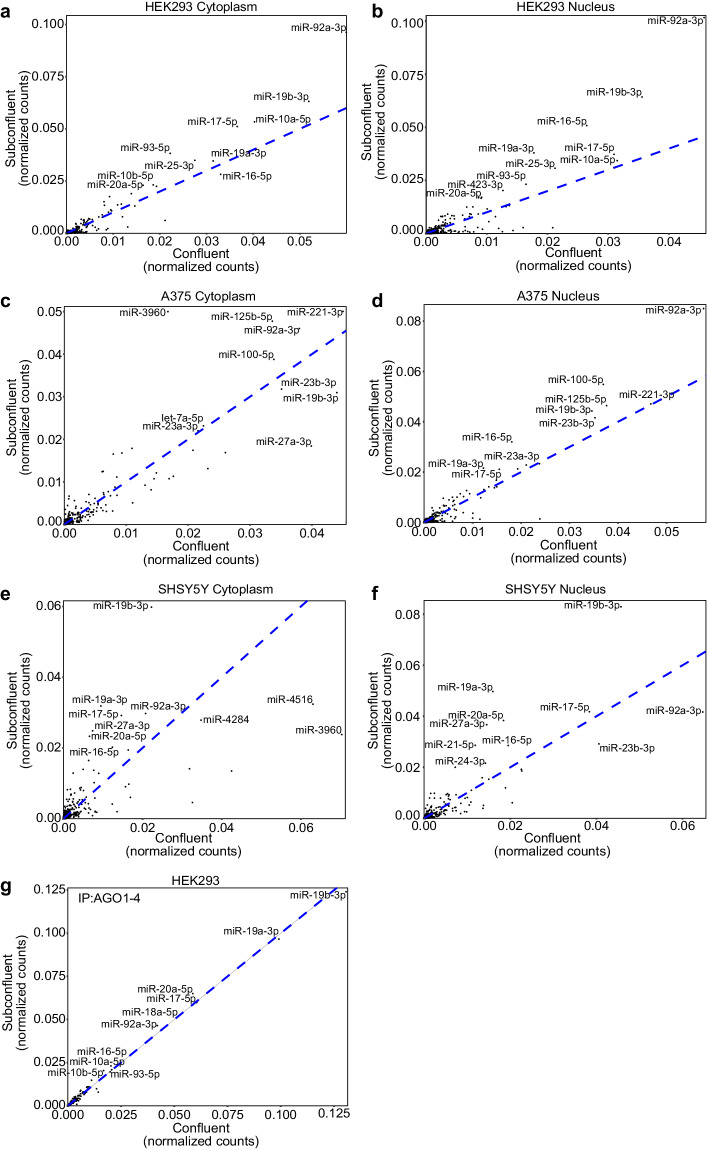
Correlation of miRNA sequencing data between subconfluent and confluent HEK293, A375 and SHSY5Y. Scatterplot of the normalized count correlation between miRNAs profile of HEK293 cells from (**a**) cytoplasmic fraction cells and (**b**) nuclear fraction. Scatterplot of the normalized count correlation between miRNAs profile of A375 cells from (**c**) cytoplasmic fraction cells and (**d**) nuclear fraction. Scatterplot of the normalized count correlation between miRNAs profile of SHSY5Y cells from (**e**) cytoplasmic fraction cells and (**f**) nuclear fraction. (**g**) Scatterplot of the normalized count correlation between miRNAs associated with AGO1-4 in subconfluent vs confluent HEK293 cells. Expression is measured as reads per million.

To assess the miRNA profile in subconfluent vs confluent conditions, RNA was extracted from the cytoplasmic and nuclear fraction of HEK293, A375 and SHSY5Y cells that were either subconfluent or confluent. The extracted RNA was taken through a small RNA library preparation and sequenced on Illumina NovaSeq 6000 platform. Furthermore, in subconfluent and confluent HEK293 cells, AGO1-4 was immunoprecipitated from whole cell lysates using the T6B peptide, which recognizes and binds to all four AGO proteins. After AGO1-4 immunoprecipitation the RNA was extracted using TRIzol and the small RNA cloned as previously reported^24^. The experiments were carried out in three replicates and the sequences were aligned to human miRNAs using bowtie2 and quantified using bedtools coverage^25^. Each sample contained between 1.5–15 million reads in total (Table S5). FastQC plots reveal the sufficient quality scores for all reads, providing robust data quality for further analysis (Fig. 5a-d). Heatmap of the correlation matrix of miRNA-seq samples shows the correlation coefficients distribution from 0.7 to 1 and highlights variations in miRNA expression profiles in confluent and subconfluent conditions in HEK293, A375 and SHSY5Y cancer cell lines (Fig. 5e-h). The principal component analysis revealed clustering of the samples according to their subcellular localization and confluency status (Fig. 5i-l). The cytoplasmic and nuclear miRNAs from subconfluent and confluent conditions in HEK293, A375 and SHSY5Y were plotted on a scatter plot, which revealed that the miRNA profile is dependent on cellular confluence (Fig. 6a-f; Table S6). miRNAs samples obtained from total HEK293 lysates after AGO1-4 immunoprecipitation our data did not show significant variation in miRNA profile between subconfluent and confluent cell conditions (Fig. 6g).

### Interactome of AGO proteins in the nucleus of confluent cells

**Fig. 7:**
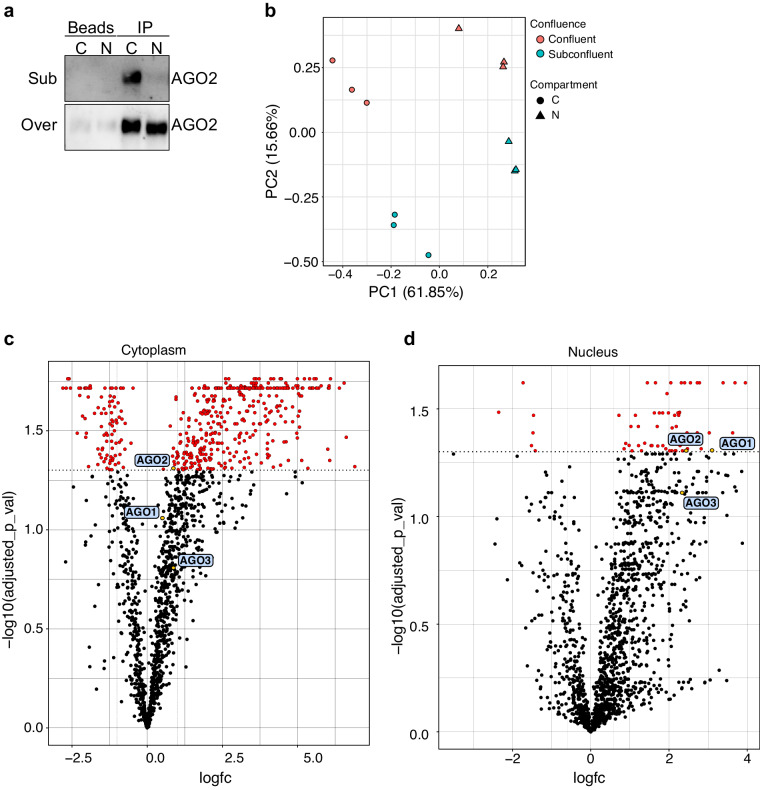
Mass spectrometry of AGO1-4 interactors in subconfluent and confluent HEK293 cells. (**a**) Representative AGO2 immunoblots from cytoplasmic and nuclear fractions of subconfluent (50%) and confluent (100%) cells after either AGO1-4 or bead only immunoprecipitation assay in HEK293 cells. (**b**) Principal component analysis (PCA) of mass spectrometry sequencing samples in HEK293. Volcano plot of mass spectrometry sequencing data obtained for (**c**) cytoplasmic and (**d**) nuclear fractions of confluent and subconfluent HEK293 cells.

To understand what the interactome of AGO proteins are in the nucleus of confluent cells, compared to non-confluent cells, we performed immunoprecipitation of AGO1-4 using the T6B peptide, from cytoplasmic and nuclear fractions of HEK293 cells. The immunoprecipitation assay indicated that AGO2 was immunoprecipitated only from the cytoplasmic fraction in subconfluent cells and from both the cytoplasmic and nuclear fraction in confluent cells (Fig. 7a). Immunoprecipitated AGO proteins were next subjected to mass spectrometry to identify protein interactors of AGO. The false discovery rate (FDR), which represents the proportion of false positive hits, among the peptide-spectrum maches (PSM), peptide groups, proteins and protein groups were less than 0.3% (Table S7). The principal component analysis revealed clustering of the experimental replicas (Fig. 7b). Enriched AGO interactors from the cytoplasmic fraction are depicted in Table S8 and from the nuclear fraction in Table S9. Fold change of 1.5 and p-value of 0.05 were considered as significant. Interestingly, in the nucleus of confluent cells AGO1-4 proteins gain a new interactome of protein partners (Fig. 7c,d).

### Supplementary information

Supplementary Table 1

Supplementary Table 2

Supplementary Table 3

Supplementary Table 4

Supplementary Table 5

Supplementary Table 6

Supplementary Table 7

Supplementary Table 8

Supplementary Table 9

## Data Availability

No custom code was used
